# In vitro chemokine (C-C motif) receptor 6-dependent non-inflammatory chemotaxis during spermatogenesis

**DOI:** 10.1186/s40659-018-0161-z

**Published:** 2018-05-22

**Authors:** Ruiying Diao, Xueyong Cai, Lu Liu, Lihua Yang, YongGang Duan, Zhiming Cai, Yaoting Gui, Lisha Mou

**Affiliations:** 1 Reproductive Medicine Centre, Shenzhen No. 2 Hospital, Shenzhen University School of Medicine, No. 3002, Sunguang West Road, Shenzhen, 518035 Guangdong China; 2 Guangdong and Shenzhen Key Laboratory of Male Reproductive Medicine and Genetics, Institute of Urology, Peking University Shenzhen Hospital, Biomedical Research Institute, Shenzhen PKU-HKUST Medical Center, Shenzhen, China

**Keywords:** CCR6, CCL20, Chemotaxis, Sertoli cell, Spermatogenesis

## Abstract

**Background:**

Chemokine (C-C motif) receptor 6 (CCR6) is present in sperm and plays a significant role in sperm motility and chemotaxis acting in the reproductive tracts. However, the expression and functional significance of CCR6 in testis are still poorly understood, especially in the process of spermatogenesis.

**Methods and results:**

CCR6 was expressed in spermatogenic cell lines and its expression was shown in an age-dependent upregulation manner from puberty to adulthood in mouse testis. Immunostaining results confirmed the localization of CCR 6 in testis. Further chemotaxis assays demonstrated that spermatogenic cells GC-1 and -2 exhibited a directional movement toward CCR6-specific ligand such as CCL20 or Sertoli cells in vitro.

**Conclusions:**

The present findings indicate that CCR6 is involved in the chemotaxis of spermatogenic cells in vitro and promotes chemotaxis under non-inflammatory conditions during normal spermatogenesis.

**Electronic supplementary material:**

The online version of this article (10.1186/s40659-018-0161-z) contains supplementary material, which is available to authorized users.

## Background

In the seminiferous epithelium, each Sertoli cell supports at least 50 germ cells and plays a crucial role during spermatogenesis. Conceivably, spermatogenesis process is involved with extensive junction restructuring particularly at the blood-testis barrier (BTB) that occurs at stages VII–VIII of seminiferous epithelial cycle [1]. Specifically, spermatogenesis is rigorously controlled by endocrine hormones and local environment. For local control, some cytokines and/or chemokines act as growth and differentiation factors in seminiferous tubule under normal physiological conditions as well as during pathophysiological states. For example, cytokines have been shown to modulate Sertoli cell transferrin release, indicating a cytokine-mediated bidirectional communication between testicular cells [2]. Evidence has shown that some cytokines and/or chemokines can potentially exert certain effects on cell populations involved in spermatogenesis and influence the post-meiotic stages of spermatogenesis [3]. For instance, rescue assays using recombinant chemokines indicated that C-C motif ligand 9 facilitates Sertoli cell chemoattraction of stem/progenitor spermatogonia, which express C-C receptor type 1 [4].

Chemokine (C-C motif) receptor 6 (CCR6) is a chemokine involved in cell interactions and leukocyte chemoattraction. It can recruit immunocytes into inflammatory tissues induced by its specific ligand chemokine (C-C motif) ligand 20, CCL20 [5]. Recent evidence indicates that CCR6 is expressed in the tail and post-acrosomal region of sperm as well as in the mid-piece and neck [6]. Appreciably, β-defensin1 (DEFB1), another ligand of CCR6, is a specific secretory polypeptide from the epididymis [6, 7]. In the testis, testicular cells can produce and secrete relatively low levels of DEFB1, which may be involved in the maintenance of sperm during the epididymis-vas deferens pass [8]. Furthermore, CCR6 may be involved in motility changes of sperm related to capacitation process, particularly in kinetic alterations and chemotaxis. Functional experiments also illuminate that CCR6 in sperm triggers Ca^2+^ mobilization stimulated by DEFB1 and CCL20, resulting in forward motility and chemotaxis of sperm in the reproductive tracts [9]. However, little is known about the constitutive expression and function of CCR6 in the testis.

## Methods

### Reagents

Two rabbit anti-CCR6 antibodies were purchased from Abcam company (ab78429, USA) and Sigma (SAB2702036, USA), respectively. Rabbit normal IgG (sc-2027), mouse monoclonal anti-occludin antibody (sc-133256) and anti-β-Tubulin antibody (sc-5274) were obtained from Santa Cruz Biotechnology (CA, USA). Recombinant Human CCL20/MIP-3α (rCCL20) was from R&D System (360-MP/CF, Minneapolis, MN, USA). Enzyme-linked immunosorbent assay (ELISA) for CCL20 was obtained from Cloud-Clone Corp. (SEA095Hu, USA). Recombinant human active DEFB1 (rDEFB1) was purchased from Abcam (ab50048).

### Cell cultures

The established lines of germ cell-1 spg (GC-1), germ cell-2 spc (GC-2), Sertoli cell origin TM4 and 15P-1, were purchased from the American Type Culture Collection (Rockville, MD, USA). GC-1 cells are characterized as being in a stage between mouse spermatocytes and spermatogonia, whereas GC-2 cells express several markers characteristic of mouse meiotic spermatocytes [10, 11]. GC-1 and -2 were cultured at 37 °C in a 5% CO_2_ atmosphere in Dulbecco’s modified Eagle’s media (11965-092; Life Technologies, Carlsbad, CA, USA) containing l-glutamine, 110 mg/L sodium pyruvate, and pyridoxine hydrochloride supplemented with 10% fetal bovine serum and 100 U/mL penicillin–streptomycin. TM4 cells were cultured at 37 °C with 5% CO_2_ in DMEM supplemented with 10% FBS, and 0.5% penicillin–streptomycin (P/S). 15P-1 cells were cultured at 32 °C with 5% CO_2_ in DMEM supplemented with 5% FBS, 1% sodium pyruvate, and 0.5% P/S.

Primary Sertoli cells (SC) were isolated from 20-day-old male mice testes and cultured in serum-free DMEM/F12 medium supplemented with EGF (2.5 ng/mL), bovine insulin (10 μg/mL), human transferrin (5 μg/mL), bacitracin (5 μg/mL) and gentamicin (20 μg/mL) at 35 °C with a humidified atmosphere of 5% CO2 in air [12]. After 1 week of culture, cell purity was established through cell morphology after vimentin immunostaining, presence of lipid inclusions active phagocytosis, and the absence of Leydig cells.

### Samples

The use of human specimens for this project was approved by the Regional Committee for Medical Research Ethics and the Human Ethics Committee of Shenzhen No. 2 Hospital and Peking University Shenzhen Hospital. Informed consent was obtained from each patient. The approval reference number is 20090018. The fertile human testis specimens were obtained from postmortem studies and orchiectomies. The specimens were andrologically examined to exclude testicular tumors by testicular ultrasound. The age range of the subjects was 24–40 years old. All of the fertile men had fathered at least one child without assisted reproductive techniques, such as IVF, ICSI, and IMSI. Men with a history of orchitis, surgery of the vasa deferentia, bilateral orchiectomy, chemotherapy or radiotherapy, obstructive azoospermia (OA), non-obstructive azoospermia (NOA), retrograde ejaculation, bilateral cryptorchidism, numerical or structural chromosome abnormalities, or Y chromosome deletions were excluded.

Male C57/6BL mice (aged 3 days, 2, 4 or 8 weeks) were purchased from the Southern Medical University Animal Center, Guangzhou, China. Animals were killed by CO_2_ asphyxiation. All animals were treated according to the Guide for the Care and Use of Laboratory Animals prepared by the Institute of Laboratory Animal Resources for the National Research Council. And the study was approved by the local ethical committee.

### Western blot analysis

Lysed proteins from GC-1 or -2 cell lines and testis tissues were centrifuged for 10 min at 12,000×*g*. The supernatant fractions were mixed with 5 × SDS sample buffer and boiled for 5 min. Each extract containing 40 µg proteins was subjected to 10% polyacrylamide gel electrophoresis and transferred onto polyvinylidene difluoride membranes (Millipore, Billerica, MA, USA). Membranes were blocked with Tris-Buffered Saline Tween-20 (TBST, pH7.5) containing 5% nonfat dry milk or 1% BSA for 3 h. Then, it was incubated overnight at 4 °C with respective antibody for CCR6 or β-tubulin, followed by corresponding HRP-conjugated secondary antibodies for 2 h at room temperature. Bound antibodies were detected using the chemiluminescence Western blot detection kit (Pierce, Waltham, MA, USA) according to the manufacturer’s recommendations. Band intensities were analyzed with the Image J software (NIH, Bethesda, MD, USA).

### Immunohistochemistry and immunofluorescence staining

Testicular tissues obtained from testis tissues were immediately stored in 4% paraformaldehyde solution after orchiectomy. After fixing and embedding, serial sections of 5 µm thickness were obtained. Paraffin sections of testis were deparaffinized using xylene, rehydrated through a graded series of ethanol, and incubated in 3% hydrogen peroxide in methanol. After antigen retrieval and blocking with 5% normal goat serum, sections were incubated with rabbit anti-CCR6 or anti-TNF-α antibody overnight at 4 °C, followed by biotin-conjugated anti-rabbit secondary antibody. DAB reaction was developed at the site of peroxidase activity. Then, sections were rinsed and washed with deionized water for 5 min, and the nuclei were counterstained with Mayer’s hematoxylin and mounted. For negative control, the primary antibody was replaced with goat serum without peptide immunization. The specimens were observed under a microscope (DMLB; Leica Microsystems, Wetzlar, Germany).

For immunofluorescence staining, sections from testes were washed three times after overnight incubation with rabbit anti-CCR6 antibody or and mouse anti-occludin antibody overnight at 4 °C. Slides were incubated with Alexa 488-conjugated goat-anti-rabbit or plus Alexa 594-conjugated goat-anti-mouse secondary antibody (1:500 dilution, Molecular Probes, Eugene, OR) at room temperature for 1 h. Under a fluorescence microscope (SPOT software, Olympus, Tokyo, Japan), ten random fields were captured for statistical analysis. Negative controls were obtained after omitting the primary antibody.

### ELISA for CCL20

Quantitative measurement of CCL20 *level* was analyzed in the culture supernatant from TM4, 15P-1 cell line or primary Sertoli cells (SC) with an ELISA-based kit following manufacturer’s instructions. The culture supernatant was centrifuged and separated at 1000×*g* for 20 min. Transwells were used for the diluted standard, blank, and sample. The luminescent signal produced from TMB substrate was measured at 450 nm using a spectrophotometer (Thermo Fisher, Waltham, MA, USA). Each sample was tested in duplicate in two separate measurements.

### Chemotaxis assays

Chemotaxis assays were performed using 24-well transwells (6.5 mm/diameter, 8 µm/pore; Costar, Corning Inc., Corning, NY, USA) as previously described [13]. Briefly, GC-1 or − 2 (1 × 10^4^/cells, 100 µL) were added to 8 µm-pore transwell inserts, coated with 25 µg of growth factor-depleted Matrigel (Becton–Dickinson Immunocytometry Systems, San Jose, CA, USA). The bottom chamber contained serum-free RPMI with or without CCL20 (50 ng/mL) or rDEFB1 (500 ng/mL) or TM4 or 15P-1 cell line or primary SC (1 × 10^4^/cells, 100 µL). For the purpose of blocking migration, each condition was prepared in a separate aliquot and incubated with anti-CCR6 antibody (ab78429 or SAB2702036). Normal rabbit IgG was used as negative control. Migration was conducted at 37 °C, 5% CO_2_ for 24 h. Migrated cells were collected from the lower compartment, centrifuged at 450×*g*, and counted using a hemocytometer. Only live cells, as determined by trypan blue exclusion, were considered in the quantification. Experiments were conducted in triplicate, and results were shown as the number of cells migrated in response to each treatment, respectively. The results for transwell assays were reported as chemotactic index (fold increase over baseline).

### Statistical analysis

The Statistical Package for the Social Sciences (SPSS Inc., Chicago, IL, USA) was used for statistical analysis. All experiments were conducted at least three times. Data were expressed as the mean ± SEM. Comparisons were subjected to one-way analysis of variance (for multi-group comparisons). Statistical significance was set at p < 0.05.

## Results

### Expression characterization of CCR6 and CCL20 in normal testis

**Fig. 1:**
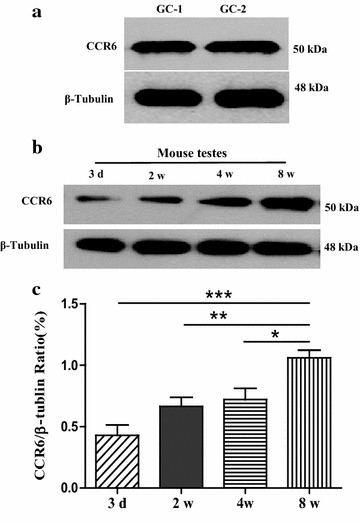
Expression characteristics of CCR6 protein in spermatogenic cell lines and mouse testis. **a** Western blots showing the expression of CCR6 in spermatogenic GC-1 and GC-2 cell lines. β-Tubulin was used as loading control. n = 5 in each group. **b** Representative western blot results showing the age-dependent up-regulated expression of CCR6 protein in mouse testes from 3 days, 2, 4 and 8 weeks age groups (n = 6 in each group). β-Tubulin was used as loading control. **c** Statistical analysis of average optical density of western blotting bands in **b**. *p < 0.05 compared with 8 weeks age group; **p < 0.01 compared with 8 weeks age group; ***p < 0.001 compared with 8 weeks age group

To investigate whether CCR6 protein was present in testis and plays an important role during spermatogenesis, the study investigated the expression of CCR6 in spermatogenic cell lines and seminiferous epithelium, respectively. Firstly, western blot results showed that CCR6 was expressed in mouse spermatogxonia GC-1 and spermatocyte GC-2 cell lines (Fig. 1a). Then, we compared the expression of CCR6 in mouse testes obtained from different age groups. As shown in Fig. 1b, c, CCR6 expression was significantly up-regulated by 59.53% (p < 0.001), 37.41% (p < 0.01) and 32.08% (p < 0.05) in adult mice (8 weeks age group, 1.06 ± 0.20) compared to 3 days, 2 and 4 weeks age groups, respectively. *Furthermore*, *the presence of CCR6 expression was also confirmed in normal adult human testis* (Additional file 1: Figure S1). Therefore, the presence of CCR6 protein in testis and its expression in an age-dependent up-regulation manner may indicate the coincidence between the spermatogenesis and CCR6 expression in the testis from puberty to adulthood.

**Fig. 2:**
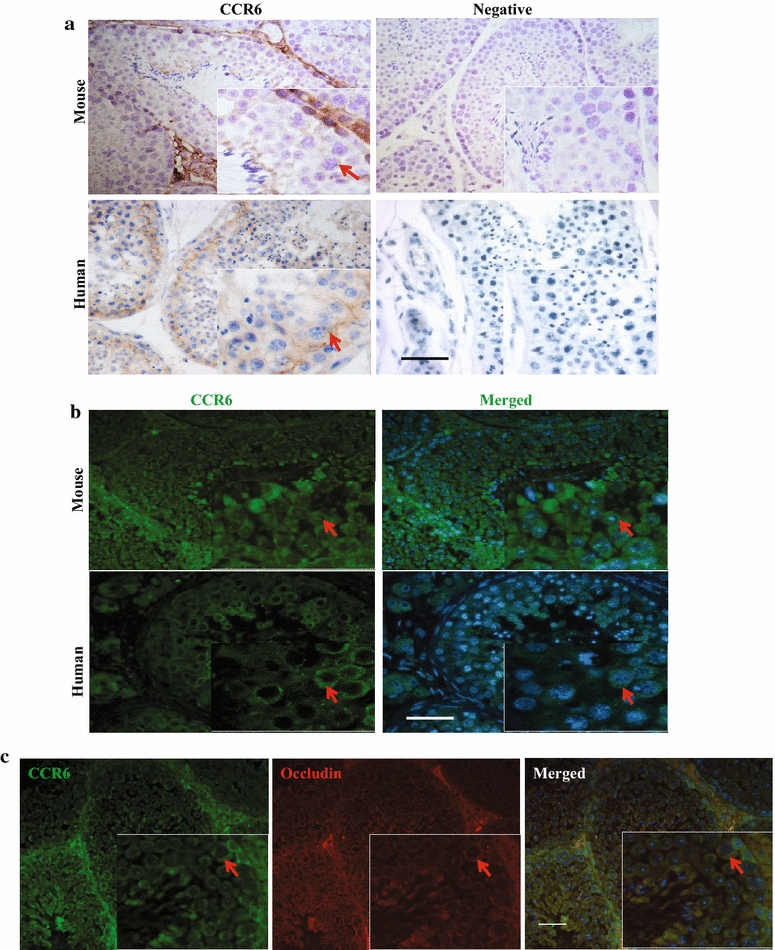
Characteristics of CCR6 localization in normal testis. **a** Representative immunohistochemical images showing the expression of CCR6 in mouse and human testes (arrow). Nuclei were counterstained with hematoxylin. Enlarged images are shown in the inset; at least three independent experiments were done. Scale bar = 50 µm. **b** Representative immunofluorescence images showing the expression of CCR6 in mouse and human testes (arrow), respectively. Nuclei were counterstained with Hoechst 33258. Enlarged images are shown in the inset; At least three independent experiments were done. Scale bar = 50 µm. **c** Representative co-staining images showing the expressions of CCR6 (green) and occludin (red) in mouse testis (arrow). Nuclei were counterstained with Hoechst 33258. Enlarged images are shown in the inset; At least three independent experiments were done. Scale bar = 50 µm

**Fig. 3:**
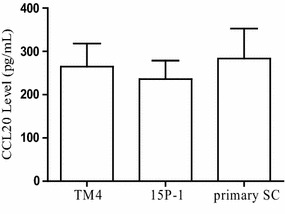
Quantitative measurement of CCL20 *level* (pg/mL) in the culture supernatant of TM4, 15P-1 cell line and primary Sertoli cells (SC). As determined by ELISA, CCL20 was present in the culture supernatant of TM4, 15P-1 cell line and primary SC

Under normal physiological states, focal inflammatory cytokines can be found in the testicular interstitium, such as TNF-α (Additional file 2: Figure S2). However, no inflammatory infiltration exists in the seminiferous epithelium due to the integrity of blood-testis barrier (BTB). Then, immunostaining data revealed that CCR6-positive signals were detected in the spermatogenic cells, spermatids and testicular interstitial area of mouse and human testes (Fig. 2a, b), respectively. The co-localization of CCR6 and occludin, a key member of tight junction strands of blood-testis barrier (BTB), suggested that CCR6 signals were localized in the cell membrane, especially in the tight junction between Sertoli and germ cells (Fig. 2c). Then, this study attempted to determine the cellular origin of CCR6-specific ligand CCL20 in the seminiferous epithelium. The data from ELISA results confirmed that CCL20 was present in TM4, 15P-1 cell line and primary SC (Fig. 3), indicating that Sertoli cells may be the main cellular origin of CCL20.

### Involvement of CCR6 in the non-inflammatory chemotaxis during normal spermatogenesis

**Fig. 4:**
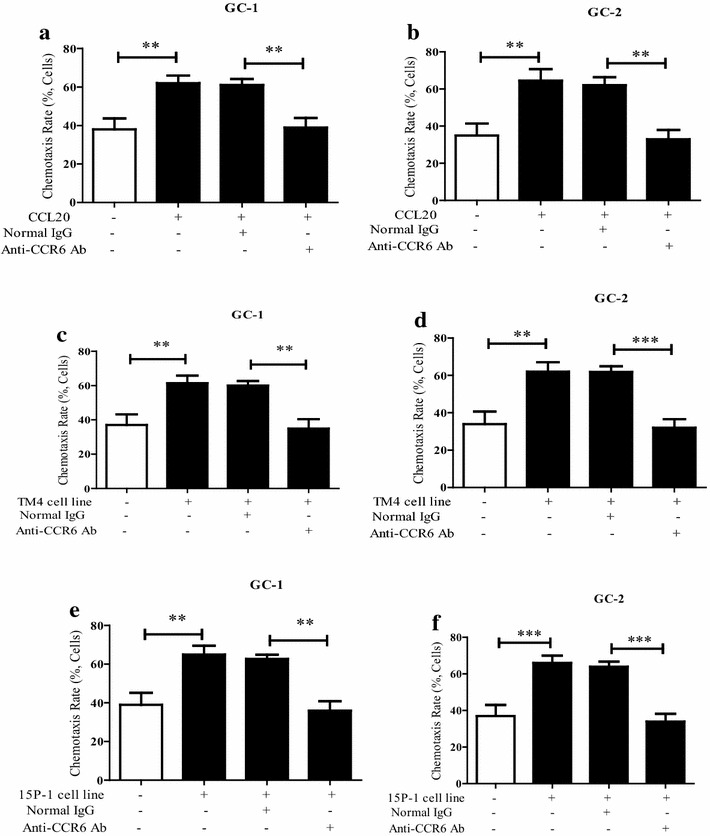
The involvement of CCR6 in the chemotaxis of mouse spermatogenic cell lines (GC-1 and -2) induced by CCL20, TM4 or 15P-1 cell line in vitro. The chemotactic activity of CCL20 to GC-1 (**a**) and GC-2 (**b**), with or without the immunodepletion of anti-CCR6 antibody. The chemotactic activity of the culture supernatant of TM4 cell line to GC-1 (**c**) and GC-2 (**d**), with or without the immunodepletion of anti-CCR6 antibody. The chemotactic activity of the culture supernatant of 15P-1 cell line to GC-1 (**e**) and GC-2 (**f**), with or without the immunodepletion of anti-CCR6 antibody. Normal rabbit IgG was used as negative control. **p < 0.01 and ***p < 0.001. n = 10 in each group

To investigate whether CCR6 is involved in the chemotaxis of spermatogenic cells, this study used rCCL20 or culture supernatant from TM4 or 15P-1 cell line as stimulus factors, respectively. The chemotaxis assay results showed that rCCL20 can significantly stimulate the chemotaxis of two cells (Fig. 4a, b). The immunodepletion of CCR6 by specific anti-CCR6 antibody (two antibodies from different company) significantly impaired cell chemotaxis compared with non-specific normal IgG, indicating that chemotaxis induction of rCCL20 was dependent on the function of CCR6 receptor on GC-1 or -2. Using conditional co-culture, chemotaxis induction by the culture supernatant from TM4 cell line can mimic the effects of rCCL20. The antagonistic effect of anti-CCR6 antibody could also be observed in this TM4 or 15P-1 cell line supernatant-induced chemotaxis acting (Fig. 4c, d). Furthermore, CCR6-dependent non-inflammatory chemotaxis was also found under the stimulation by rDEFB1 or culture supernatant from primary SC (Additional file 3: Figure S3). Therefore, these in vitro functional results further confirmed that CCR6 may play an important role in the chemotaxis of spermatogenic cells. That is, Sertoli cells produced CCL20, which can induce GC-1 or -2 non-inflammatory chemotaxis through the receptor CCR6.

## Discussion

Spermatogenesis is a complex process mediated by local growth or differentiation factors and a compartmentalized process sequestered within the BTB [14, 15]. Increasing amounts of evidence indicates the non-inflammatory roles of growth factor and cytokines in development and differentiation of germ cells during spermatogenesis [16, 17]. Sertoli cells are involved in the transduction of signals using testicular receptor to regulatory elements of several responsive genes under non-inflammatory autocrine/paracrine states [16]. Previous studies reported that chemotaxis acting on sperm after ejaculation from the epididymis depends on CCR6 in sperm, primarily improving forward motility and promoting sperm-egg fertilization [6, 9]. CCR6 in human and mouse sperm mostly originates from spermatogenic cells, although it is possible that CCR6 is contributed to the sperm by epididymosomes [18, 19]. Recent research has also pointed to a putative role for CCR6/CCL20 axis in optimal germinal centre (GC) kinetics and B memory cell function [20, 21]. Then, is CCR6, as the receptor of CCL20, involved in the process of spermatogenesis?

As for the presence of CCR6 in testis, mouse Ccr6 mRNA was previously reported to express in testis [6]. In the present study, it was further confirmed by the presence of CCR6 proteins both in spermatogenic cell lines and testis tissues. Then, we found that CCR6 was mainly expressed and localized in spermatogenic cells, such as spermatogonia, spermatocytes and spermatid cells. Furthermore, CCR6 signals were also detected in the tight junctions. After confirming the presence of CCR6, the ELISA results elucidated that CCL20 was present in the culture supernatant of TM4, 15P-1 cell line and primary SC. At the same time, the age-dependent up-regulation of CCR6 expression in mouse testis from puberty to adulthood, indicated the coincidence between CCR6 expression and essential role of CCR6 chemotaxis during normal mouse spermatogenesis.

In the seminiferous epithelium, non-lymphoid cells such as Sertoli cells, which can secrete cytokines, may be involved in germ cell-germ cell interactions in a stage-specific manner during spermatogenesis [22]. For example, IL-6, which is produced by Sertoli cells [23], is a stage-specific paracrine regulator of the seminiferous epithelium exerting specific inhibitory action on meiotic DNA synthesis [24]. Accordingly, the local chemokine cycles are important for spermatogenesis [18]. Then, what is the role of CCL20-CCR6 in spermatogenesis? Previous studies showed that CCL20 is involved in the non-inflammatory chemotaxis of sperm; thus, the present study focused on this during spermatogenesis. The in vitro chemotaxis experiments indicated that chemotaxis of CCL20 (50 ng/mL) may be involved in the migration of GC-1 and -2, and this process can be significantly inhibited by CCR6-specific antibody. Using two CCR6-specific antibodies from different commercial companies, this antagonistic effect can be achieved. At the same time, Sertoli cell-conditioned medium can induce the non-inflammatory chemotaxis of GC-1 and -2, which can be obviously inhibited by CCR6-specific antibody. This finding indicates that non-inflammatory chemotaxis of GC-1 and -2 induced by Sertoli cell-conditioned medium also needs the participation of CCR6 in germ cells. The further results also suggest that the migration of GC-1 and -2 can be stimulated by rDEFB1, which can also be significantly inhibited by CCR6-specific antibody (Additional file 3: Figure S3). Therefore, we provide evidence that the involvement of Sertoli cells and CCR6-it ligands (CCL20 or DEFB1) interaction in the migration of spermatogenic cells which is a relevant process during spermatogenesis.

Accordingly, Sertoli cells provide germ cells with the appropriate mitogens, differentiation factors, and energy sources, as well as protect them from harmful agents and from the host’s own immune system. Specifically, chemokine ligands via their receptors were required for the maintenance of mouse spermatogonial stem cells. For example, CXCL12-CXCR4 signaling is required for the maintenance of mouse spermatogonial stem cells [25]. Macrophage inflammatory protein-1 alpha is a regulator of mitotic and meiotic DNA synthesis during spermatogenesis [26]. A previous study demonstrated that CCL20/CCR6 are involved in the metastasis of a variety of tumors, including prostate, colorectal and lung cancer [27, 28]. As determined by protein chip analysis, several chemokines (including some that act through CCR6, such as CCL20 and protein hormones) were present in human follicular fluid, endometrial secretions, and seminal plasma. CCL20/CCR6 interactions can lead to proliferation and migration dependent in part on extracellular signal-regulated kinase (ERK) phosphorylation during lung adenocarcinoma growth [28]. In vitro Sertoli cells can produce and secrete CCL20. In this study, the chemotaxis assays demonstrated that spermatogenic GC-1 and -2 cell lines exhibited a directional movement toward CCL20 or Sertoli cells in vitro. This in vitro study using GC-1 and -2 cell lines confirmed that CCR6 was involved in the non-inflammatory chemotaxis of germ cells when activated by its ligand CCL20. These findings suggest that CCR6-CCL20 interaction between spermatogenic and Sertoli cells play essential roles in spermatogenesis.

## Conclusion

In conclusion, CCR6 for a receptor of CCL20 appears to be important throughout germ cell maturation, development and migration. Blockade or loss of the receptor results in the inability of cell chemotaxis of spermatogenic cells to CCL20 or Sertoli cells. Therefore, the present findings further support an essential role of CCR6 in non-inflammatory chemotaxis, which is conducive to the inward migration of germ cells during the seminiferous epithelial cycle.

## Additional files

**Additional file 1: Figure S1.** Expression of CCR6 in normal adult human testis. Representative western blot results showing the expression of CCR6 in normal adult human testis (n = 5). β-Tubulin was used as loading control.

**Additional file 2: Figure S2.** Characteristics of TNF-α localization in mouse testis. Representative immunohistochemical images showing the location of TNF-α in the testicular interstitial of mouse testis (arrow). Nuclei were counterstained with hematoxylin; At least three independent experiments were done. Scale bar = 50 µm.

**Additional file 3: Figure S3.** The involvement of CCR6 in the chemotaxis of mouse spermatogenic cell lines (GC-1 and -2) induced by rDEFB1 or primary sertoli cells in vitro. The chemotactic activity of rDEFB1 to GC-1 (A) and GC-2 (B), with or without the immunodepletion of anti-CCR6 antibody. The chemotactic activity of the culture supernatant of primary Sertoli cells to GC-1 (C) and GC-2 (D), with or without the immunodepletion of anti-CCR6 antibody. *p < 0.05, **p < 0.01 and ***p < 0.001. Normal rabbit IgG was used as negative control. n = 10 in each group.
