# Supplementary figures and images for: In vitro chemokine (C-C motif) receptor 6-dependent non-inflammatory chemotaxis during spermatogenesis
Source: Biol Res. 2018 May 22;51:12. doi: 10.1186/s40659-018-0161-z (PMC5963036; doi:10.1186/s40659-018-0161-z)

## Additional file 1: Figure S1

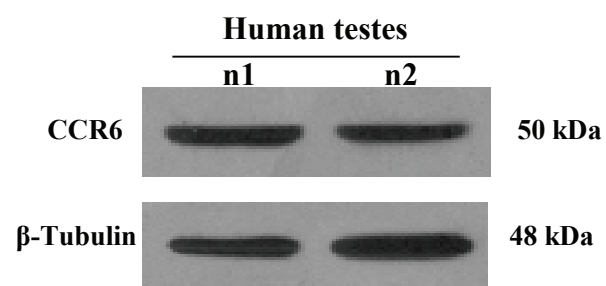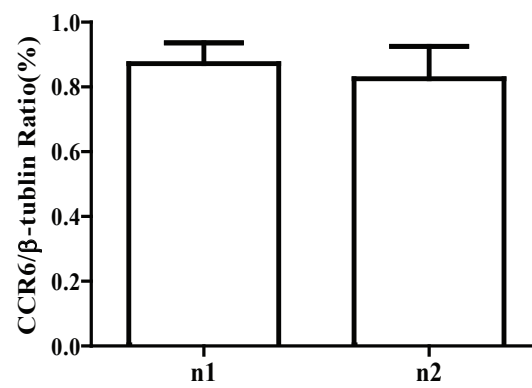

Supplement: Supplementary file 1 — Additional file 1: Figure S1. Expression of CCR6 in normal adult human testis. Representative western blot results showing the expression of CCR6 in normal adult human testis (n = 5). β-Tubulin was used as loading control. [file 40659_2018_161_MOESM1_ESM.pdf]

## Additional file 2: Figure S2

TNF- $\alpha$

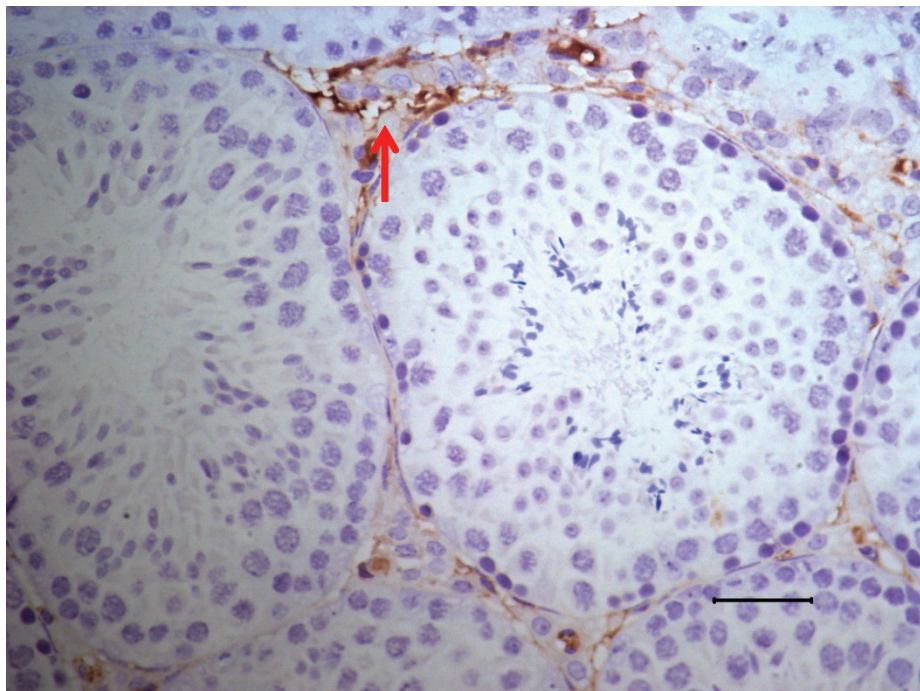

Supplement: Supplementary file 2 — Additional file 2: Figure S2. Characteristics of TNF-α localization in mouse testis. Representative immunohistochemical images showing the location of TNF-α in the testicular interstitial of mouse testis (arrow). Nuclei were counterstained with hematoxylin; At least three independent experiments were done. Scale bar = 50 µm. [file 40659_2018_161_MOESM2_ESM.pdf]

Additional file 3: Figure S3

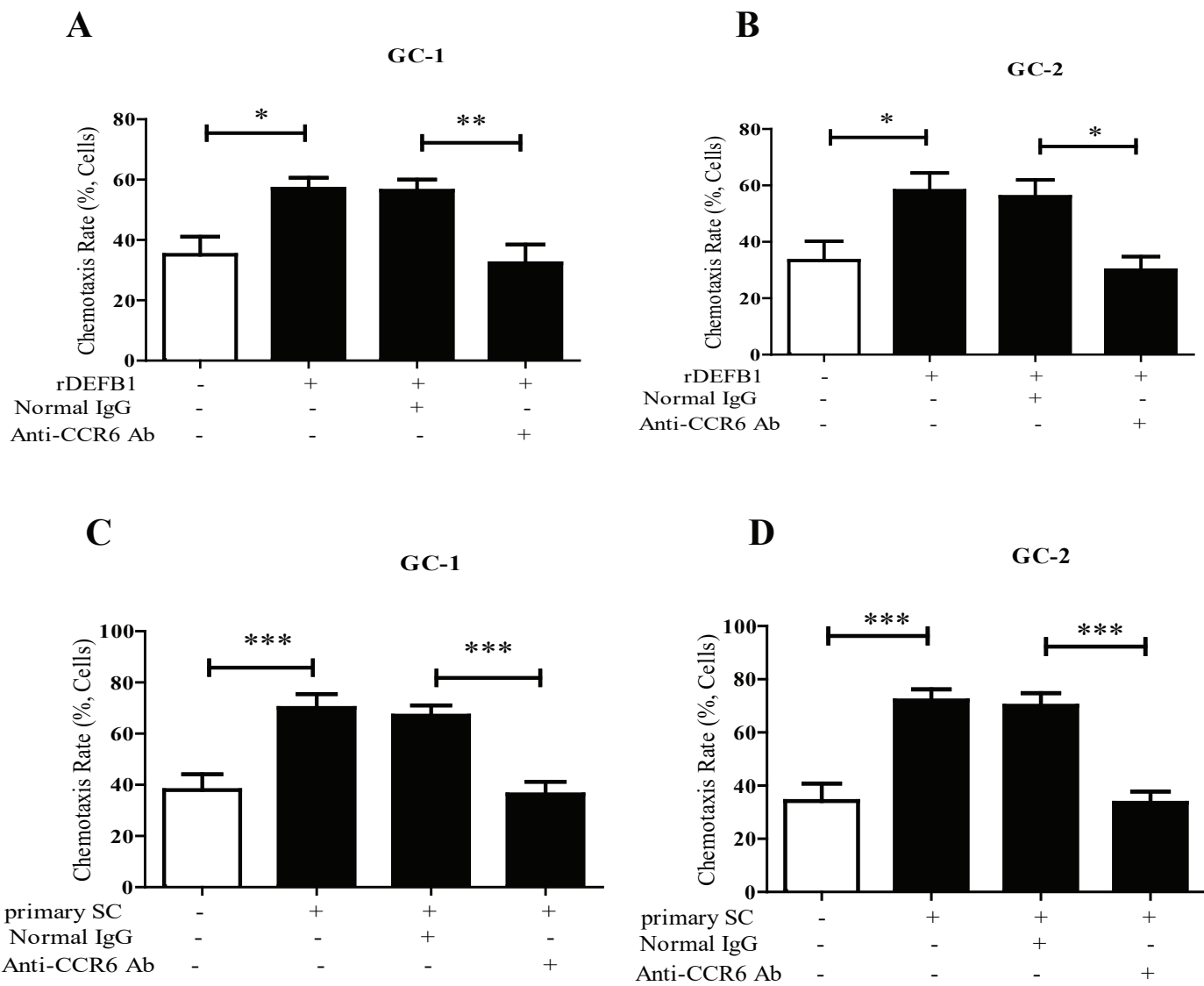

Supplement: Supplementary file 3 — Additional file 3: Figure S3. The involvement of CCR6 in the chemotaxis of mouse spermatogenic cell lines (GC-1 and -2) induced by rDEFB1 or primary sertoli cells in vitro. The chemotactic activity of rDEFB1 to GC-1 (A) and GC-2 (B), with or without the immunodepletion of anti-CCR6 antibody. The chemotactic activity of the culture supernatant of primary Sertoli cells to GC-1 (C) and GC-2 (D), with or without the immunodepletion of anti-CCR6 antibody. *p < 0.05, **p < 0.01 and ***p < 0.001. Normal rabbit IgG was used as negative control. n = 10 in each group. [file 40659_2018_161_MOESM3_ESM.pdf]
